# In vitro biocompatibility testing of some synthetic polymers used for the achievement of nervous conduits

**Published:** 2011-08-25

**Authors:** R Mihai, IP Florescu, V Coroiu, A Oancea, M Lungu

**Affiliations:** * ‘Bagdasar Arseni’ Clinical Emergency Hospital Romania; ** National Institute of Research and Development for Biological Sciences, Bucharest Romania

**Keywords:** Polyvinyl alcohol, polyethylenglycol, polyvinyl chloride, dermal fibroblasts, MTT test

## Abstract

Biocompatible synthetic polymers are largely used in the bio–medical domain, tissue engineering and in controlled release of medicines. Polymers can be used in the achievement of cardiac and vascular devices, mammary implants, eye lenses, surgical threads, nervous conduits, adhesives, blood substitutes, etc. Our study was axed on the development of cytotoxicity tests for 3 synthetic polymers, namely polyvinyl alcohol, polyethylene glycol and polyvinyl chloride. These tests targeted to determine the viability and morphology of cells (fibroblasts) that were in indirect contact with the studied polymers. Cell viability achieved for all the studied synthetic polymers allowed their frame in biocompatible material category. Cell morphology did not significantly change, thus accomplishing a new biocompatibility criterion. The degree of biocompatibility of the studied polymers varied. Polyvinyl alcohol presented the highest grade of biocompatibility and polyvinyl chloride placed itself at the lowest limit of biocompatibility. The results achieved allowed the selection of those polymers that (by enhancing their degrees of biocompatibility due to the association with various biopolymers) will be used in the development of new biocompatible materials, useful in nervous conduits manufacture.

## Introduction

Polymers are organic materials, composed of a large number of macromolecules, which in fact form covalent atom bindings. Due to the covalent nature of intermolecular bindings, the electrons are located between the atoms and consequently the polymers tend to have low thermal and electric properties. Polymer thermal and mechanic conduct is influenced by the chemical composition, polymer chain structure and the atomic weight of the molecules. The number of polymer biomaterials is quite high. They differ by monomer chemical nature, polymerisation grades and conditions, molecular weight, steric structure, additives and the presence of foreign bodies [1]. By considering tissue reactions of all implant materials as foreign body reactions, the destination of a certain material is determined by the intensity of the interactions and irritability capacity [2–4]. 

The biocompatibility of a material is defined as the material property, which, after implantation in a living organism, does not produce adverse reactions and is accepted by the adjacent tissues. Thus, the implant material must not present toxicity and must not induce inflammatory reactions [5,6]. Implant biocompatibility depends on numerous factors, such as the patient's health status, age, tissue permeability, immunological factors and implant characteristics (material roughness and porosity, chemical reactions, corrosion properties, toxicity) [7].

Biocompatibility estimation imposes the knowledge of the irritative effect, systemic toxicity level, allergic effect, material stability in the physiologic medium and the possible interactions between cell constituents and the implanted material [8]. The testing program must be chosen depending on polymer nature, implant form, implant location and the length of time for implant keeping in the organism. The testing program includes three stages, respectively preclinical testing, animal implantation and clinical testing on human subjects. Biocompatibility testing is a sine qua non condition of biomedical polymer use. The great difficulty resides in the burden of colligation between polymer supra molecular structure and organism physiological medium, considering cell and sub cell reactions and hystochemical, physical and chemical   aspects of implant accessibility or reject [9]. 

	A basic request of biomedical engineering is the examination of foreign body reactions. Generally it is encountered a tissue reaction to implant even if the material is chemically inert. 

Medical evaluation of the polymer must be preceded by tissue and blood compatibility assessment and by in vitro and in vivo tests, individualized for each polymer [10]. 

Although in vivo experiments are much more conclusive, due to higher costs and laborious techniques, there are mainly used a series of in vitro tests [11]. 

The goal of our study was to evaluate the in vitro biocompatibility of some synthetic polymers (Polyvinyl alcohol–APV, Polyethylene glycol–PEG and polyvinyl chloride–PVC), by using fibroblast cultures in order to use them for the achievement of nervous conduits. In neural tissue engineering, degradable materials are preferred whenever possible due to their low incidence of inflammation and scar–very deleterious for the nerve function. The lack of biocompatibility and bioactivity of some synthetic polymers prevents them from promoting cell attachment, proliferation, and differentiation [12,13].  In the very specific case of nerve conduits, whatever its form of a biologic tube, synthetic tube or tissue–engineered conduit, it should facilitate neurotropic and neurotrophic communication between the proximal and distal ends of the nerve gap, block external inhibitory factors, and provide a physical guidance for axonal growth [14]. The management of peripheral nerve injury requires a thorough understanding of the complex physiology of nerve regeneration, with the ability to perform surgery under magnification [15]. Severe nerve injuries that led to nerve gaps can be surgically repaired either by autografts or nerve guide conduits. Artificial nerve guide conduits have the advantage over autografts due to their availability and ease of fabrication. However, clinical outcomes associated with the use of artificial nerve conduits are often inferior to that of autografts, particularly over long lesion gaps [16].

Although autografts are the gold standard in nerve gap repair, they have a series of drawbacks. In order to obviate them, some authors reported the use and test of ultrathin microporous biodegradable PCL and PCL/PLA films for their compatibility with motor neuron–like NG108–15 cells and primary Schwann cells. Immunohistochemical staining showed that regenerated nerve tissue and penetrated Schwann cells have the potential to span the whole length of the conduit in 2 weeks [17].

In humans, the studies are rather limited to short gaps but quite encouraging for sensitive nerve, proving that collagen conduits are an effective treatment for post–traumatic painful neuromas of digital nerves and common digital nerves [18].

## Methods

### Polymeric materials

PVC for medical use has relative molecular mass of 25000 Da and a polymerization degree of 70; it was obtained by polymerization in suspension (OLTCHIM Ramnicu Valcea, Romania). APV has relative molecular mass of 71000 Da and a polymerization degree of 1600 and it was obtained from ROMACRIL Rasnov SA, Romania. PEG is from SERVA and it has relative molecular mass of 4000 Da and a polymerization degree of 70.

### Cell culture testing

PVC, APV and PEG in vitro biocompatibility was evaluated by fibroblast culture testing (MRC5 – human lung fibroblasts). The culture medium was represented by DMEM, supplemented with a 10% foetus calf serum and a 3 antibiotic mix: penicillin, streptomycin and neomycin.

### Cell proliferation determination

The cytotoxic effect of polymeric materials was measured by the MTT test.

MTT test (3–4,5–dimethylthiazol–2–yl)–2,5–diphenyltetrazolium bromide) is based on tetrazolium soluble salt conversion in formazan with the help of mytocondrial NAD and NADH–dehydrogenases from the viable cells.

In order to achieve the PVC, APV and PEG extracts, samples from each polymer (sterilized by an 8 hour UV radiation exposure) were immersed in a DMEM medium, supplemented with a 10% foetus calf serum, for 24 hours, at an extraction report of 6 cm^2^ sample/1 ml medium (PVC and APV) and 3 mg PEG/1 ml medium.

For the experiment, the cells were seed at a 2,5x104 cell/ml density in 24 well plates and incubated in a humid atmosphere with 5% CO2, at 370C, for 24 hours. After incubation, the culture medium was replaced with the medium that contained the polymer extracts. After 24, respectively 48 hours of culture within the polymer extracts, we added the MTT reactive. After 3 hours of culture in the dark, the formed formazan crystals were made soluble by isopropanol and the absorbance in the holes was measured at 570 nm.

The percent of viable cells cultured with the polymer extracts was calculated by reporting to a control sample (cells cultured without the extract) considered as having a viability of 100%.

### Cell morphology determination

Qualitative biocompatibility analysis was done by comparing the morphology of MRC5 fibroblast cultured with the PVC, APV and PEG samples to control cell cultures.

For cell morphology analysis, cells cultured with the polymer extracts for 48 hours were washed with PBS, fixed with methanol at –20 degrees C, colored with Giemsa solution according to von Gieson method, visualized at a Zeiss inversed microscope and then photos were taken. 

## Results 

The biocompatibility of PVC, APV and PEG was evaluated in vitro by measuring the mitochondrial dehydrogenase activity (MTT test) and by the analysis of cell morphology of the fibroblasts cultured in indirect contact with these synthetic polymeric materials (extract samples). Experiments were carried in a cell culture laboratory accredited by RENAR in accordance with SR EN ISO 17025:2005 standard requirements to determine the cytotoxicity of the medical devices by quantitative and qualitative methods.

**Figure 1:**
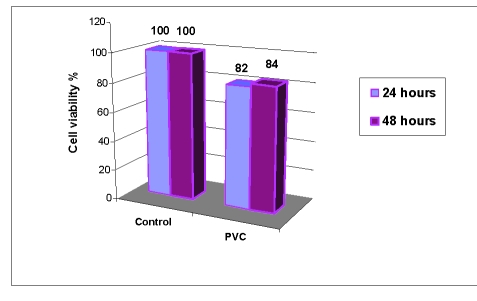
Fibroblast viability in presence of PVC extract after 24 and 48 hours of culture

The results for cell proliferation with MTT test for PVC are presented in Figure 1.

The results were based on MTT absorption and reflected mitochondrial activity in the viable cells. A cell viability percent higher than 80% signified a good biocompatibility for the tested polymer–PVC.

Cell morphology analysis was realized by comparing the morphology of fibroblasts that were cultured with PVC samples to the control cells.

**Figure 2:**
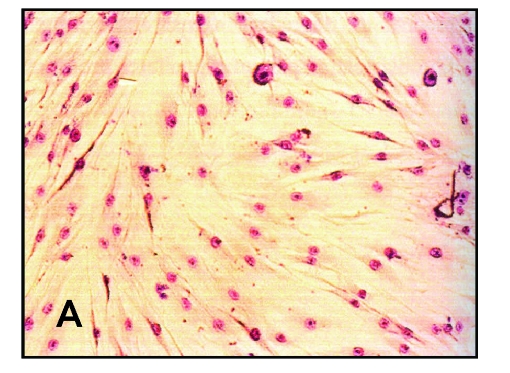
Light microscopy images of cell cultures after 24 hours. A –control sample; B – PVC sample

The control fibroblasts presented a normal phenotype. The cells had a stellate or elliptic form, with a normal colored nucleus, 2–3 prominent nucleoli and the cytoplasm was granulated. The fibroblasts cultured with the PVC samples remained normal after the culture period, demonstrated by light microscopy at 24 and 48 hours (Figure 2).  

**Figure 3:**
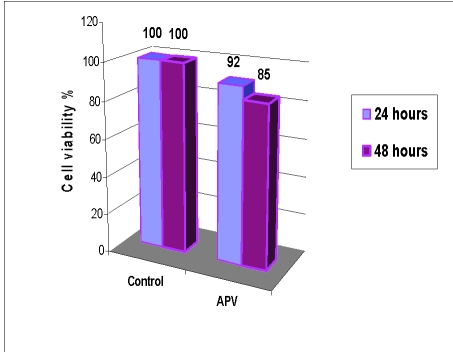
Cell proliferation test (MTT) at 24 and 48 hours in the presence of APV extract

These tests confirmed the fact that the analyzed PVC sample did not present cytotoxic effect and did not inhibit cell proliferation, having a satisfactory degree of biocompatibility as compared to the control group. The results of MTT test for APV are presented in Figure 3.

Cell viability was the highest for those ones incubated with the APV extract (over 85%), as compared both to the control sample and the other polymers samples tested. 

**Figure 4:**
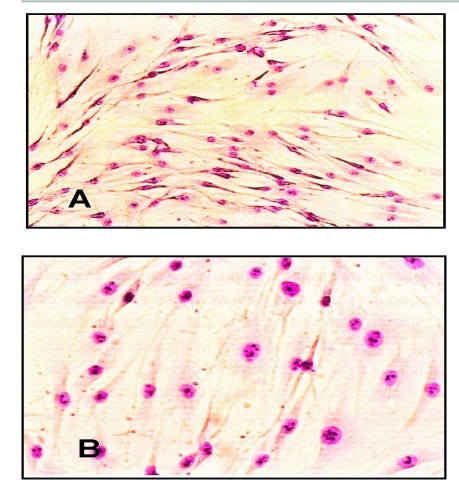
Light microscopy images of cell cultures after a 48 hours period of culture with the APV extract (B) and without it (control sample – A).

For cell morphology analysis, the images taken for the APV sample were compared to the control images (cell culture without the polymer) (Figure 4). The analysis of cell morphology showed that the cells incubated with the APV extract did not exhibit different aspects, fibroblasts presenting, in their vast majority, a normal phenotype. 

**Figure 5:**
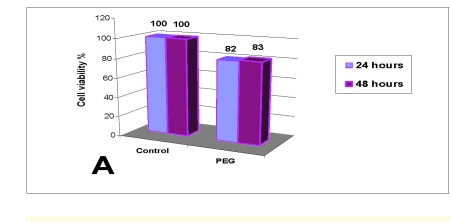
Cell proliferation test (MTT) at 24 and 48 hours

APV biocompatibility tests showed that this polymer had a good degree of biocompatibility, because it did not inhibit cell proliferation and did not lead to morphology changes for a significant number of cells. In Figure 5 we present the degree of cell proliferation for PEG, at 24 and 48 hours, evaluated by MTT test.

Cell viability was lower for those ones incubated with the PEG extract, as compared to the control, but it is higher than 80% after both periods of time. 

**Figure 6:**
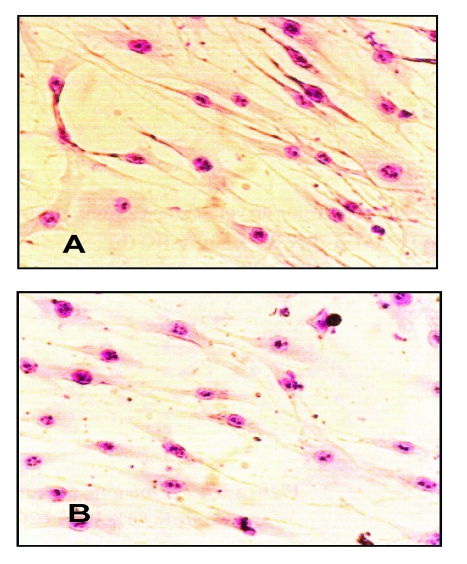
Light microscopy images of cell cultures after 48 hours for PEG extract (B) and for the control sample (A).

The results achieved for PEG showed a good in vitro degree of biocompatibility, slightly lower than the control samples

## Discussions

The biocompatibility of 3 synthetic polymers was tested: Polyvynilic alcohol (APV), Polyethylene glycol (PEG) and Vynil polychloride (PVC). PVC is one of the mainly used medical materials due to its multiple properties: flexibility, bending resistance, compactness, chemical and biological resistance, gamma irradiation, ethylene oxide and autoclave sterilization. 

APV presents a special interest due to its characteristics that recommends it for numerous medical and pharmaceutical applications. Its long lifetime in the organism is due to the lack of tissue disintegration. APV multiple uses, including peripheral nerve surgery, is based on its physical and chemical properties: water solubility, thermical stability, film making capacity, stretching resistance, organic solvents and oil  resistance, good biodegradability, nontoxicity  and non carcinogenicity. 

PEG is the most important commercial synthetic polymer. It is achieved by suspension polymerization of ethylene oxide and is purchased under liquid or solid form, with a molecular weight between 300 and 10 000 000 g/ml. The polymerization initiator induces these polymers, in different forms. PEG has a low toxicity and thus a large applicability in clinical and pharmaceutical domain. Recent studies emphasized PEG use for gene therapy vectors encapsulation. PEG hydrogels are tolerated in CNS, also. It was demonstrated that neural precursor cells were able to be photoencapsulated and cultured on the PEG gels with minimal cell death. The hydrogel blocks inflammatory and other inhibitory signals from surrounding tissue and is able to serve as a scaffold for axon regeneration [12].

The in vitro tests for the above three polymers were performed on a stabilized fibroblast culture (MRC5). There were evaluated cell viability and morphology for those cells that were in indirect contact with the studied polymers. 

These tests concluded that although all the three tested synthetic polymers were biocompatible, their degrees of biocompatibility varied. Cell viability was over 80% for all studied synthetic polymers. The degree of proliferation was the highest for the cells incubated in the presence of APV sample (over 85%), as compared to the other polymers samples tested (PVC, PEG). Thus, we could frame them all in the biocompatible material category. Cell morphology did not significantly change: this represented also a biocompatibility criterion. 

After establishing the biocompatibility, the next step is represented by studies regarding the association, by covering or mixing, of biopolymers with the analysed synthetic polymers in order to achieve new biomaterials with application in peripheral nerve surgery for nervous conduits manufacture. Besides new biomaterials, of interest is also the development of novel conduit architectural designs using porous and fibrous substrates [16]. Another beneficial influence on peripheral nerve regeneration was demonstrated to be accomplished by multichannel guidance conduits due to the limited dispersion that did not decreased the quantitative results of regeneration [19].

Despite the advancements in the field of tissue engineering, the level of functional improvement after peripheral nerve injuries did not increased. However, there are emerging hopes for the next generation of nerve conduits with the advancement of nanotechnology [15].
